# Recent advances and application value of circRNA in neuroblastoma

**DOI:** 10.3389/fonc.2023.1180300

**Published:** 2023-04-06

**Authors:** Ke Wu, Juan Tan, Chao Yang

**Affiliations:** ^1^ Department of Pharmacology, Chongqing Key Laboratory of Biochemistry and Molecular Pharmacology, Chongqing Medical University, Chongqing, China; ^2^ Child Healthcare Department, Children’s Hospital of Chongqing Medical University, Chongqing, China; ^3^ Department of Pediatric Surgical Oncology, Ministry of Education Key Laboratory of Child Development and Disorders, National Clinical Research Center for Child Health and Disorders, China International Science and Technology Cooperation Base of Child Development and Critical Disorders, Chongqing Key Laboratory of Pediatrics, Children’s Hospital of Chongqing Medical University, Chongqing, China

**Keywords:** circRNA, neuroblastoma, pathogenesis, biomarkers, tumor targeted therapy

## Abstract

Neuroblastoma (NB) is children’s most prevalent solid malignant tumor, accounting for 15% of childhood cancer mortality. Non-coding RNA is important in NB pathogenesis. As a newly identified non-coding RNA, abnormal regulation (abnormal up-regulation or down-regulation) of the circRNAs expression is implicated in the tumorigenesis of various tumors, including NB. CircRNAs primarily regulate the expression of microRNA (miRNA) target genes by microRNA (miRNA) sponge adsorption. Clinical evidence suggests that the expression of certain circRNAs is associated with the prognosis and clinical features of NB and hence may be exploited as a biomarker or therapeutic target. This review examines circRNAs that have been demonstrated to play a function in NB.

## Introduction

Neuroblastoma (NB) is the most prevalent extracranial malignant solid tumor in children, accounting for 8–10% of all malignant tumors in children and 15% of all childhood cancer deaths (1). This tumor develops from primitive sympathetic crest cells and is characterized by various clinical manifestations. It can be a benign tumor or widely metastasized (2). The adrenal gland is the most common primary site, accounting for about half of all tumors. Other tumor sites include the retroperitoneum, posterior mediastinum, neck, and bone cavity (2). Most children are diagnosed in infancy, and the five-year survival rate of children diagnosed before one year is greater (3). Available treatment methods include chemotherapy, surgery, radiotherapy, autologous stem cell transplants, and immunotherapy. Despite adopting several therapeutic techniques, the five-year survival rate for children with neuroblastoma in the high-risk group is still approximately 50%. Studying the potential pathogenesis of neuroblastoma is of great significance for early diagnosis, scientific staging, and precise treatment. Several investigations have demonstrated that gene modifications in regulating the cell cycle, cell proliferation, and programmed cell death contribute to the pathogenesis of the disease. There are numerous epigenetic factors involved in gene expression regulation, including DNA and RNA methylation, histone modification, non-coding RNAs (including long-chain non-coding RNAs (lncRNAs), microRNAs (miRNAs), and circular RNAs (circRNAs)). These epigenetic factors may directly affect tumor initiation and progression (4). Numerous research has examined the role of lncRNAs and miRNAs in the occurrence and progression of NB. The role of circRNAs in NB has garnered increasing attention in recent years.

**Figure 1:**
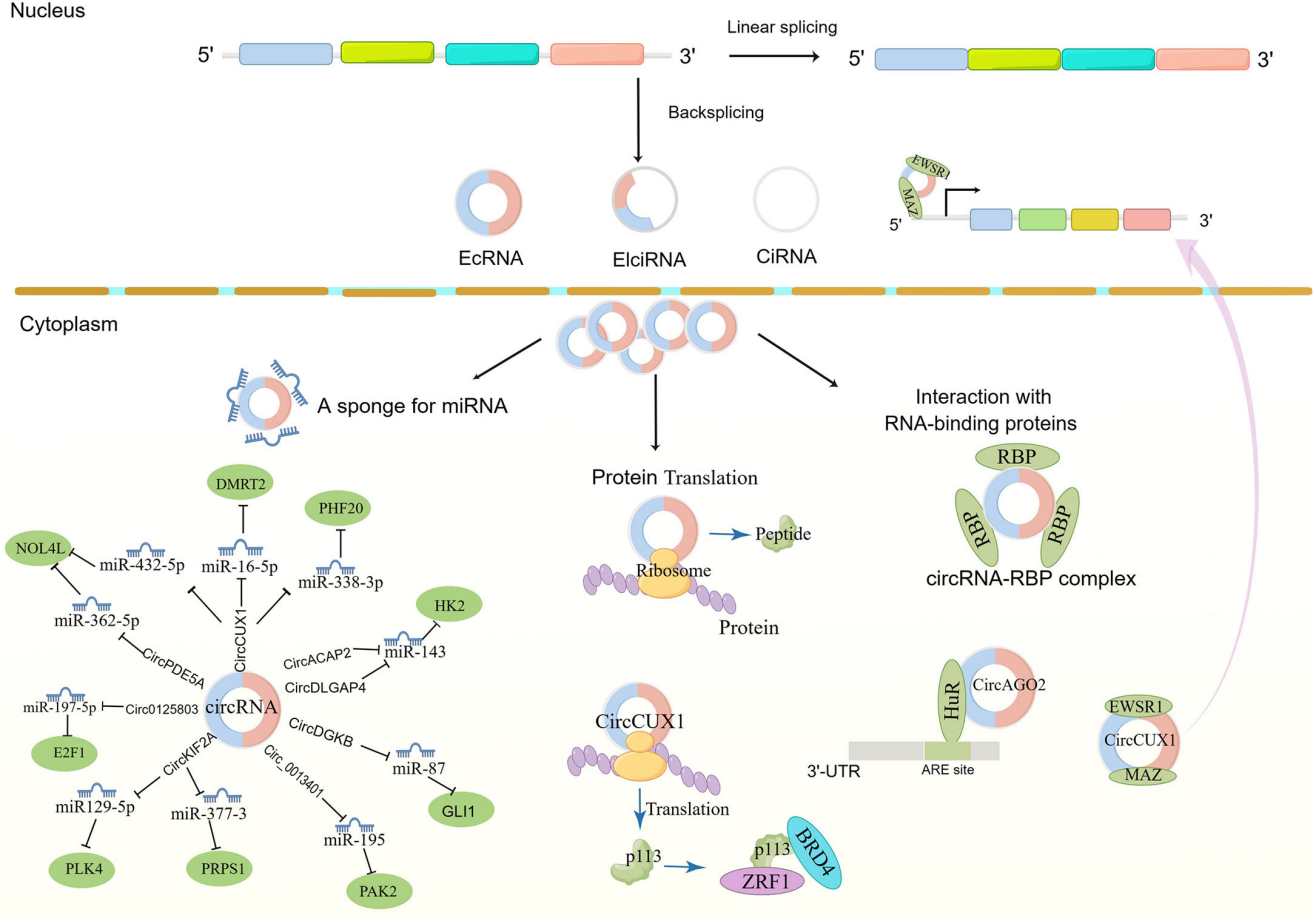
The biogenesis of circRNAs and mechanism in neuroblastoma cancer cell progression. EcRNAs, Exonic circRNAs; EIciRNAs, Exonic-intronic circRNAs; CiRNAs, intronic circRNA. EWSR1,EWS RNA binding protein 1; MAZ, MYC-related zinc finger protein; DMRT2, Doublesex and mab-3 related transcription factor 2; NOL4L, Nucleolar protein 4 like; PHF20, PHD Finger Protein 20; Zuotin-related factor 1; BRD4, bromodomain protein 4; PLK4, Polo-like kinase 4; PRPS1, Phosphoribosyl pyrophosphate synthetase 1; GLI1, Glioma-associated oncogene 1; AGO2, Argonaute 2; HK2, hexokinase 2; PAK2, P21-activated kinase 2; HuR, Human antigen R.

Non-coding RNA is believed to regulate gene expression in eukaryotic cells. Recently, it was shown that circRNAs serve a critical function in regulating gene expression in eukaryotic cells. Despite being single-stranded, cyclic RNAs have two ends that combine to form cyclic polynucleotide chains. They have superior stability over miRNAs and lncRNAs because of their circular nature, which makes them resistant to exonucleases (5). Up to now, several types of circRNAs have been identified according to their distribution and biogenesis (**Figure 1**): exonic circRNAs (EcRNAs), intronic circRNAs (ciRNAs), and exon-intron circRNAs (EIciRNAs). Research has shown that circRNAs importantly regulate gene expression. It can alter transcriptional and translational modifications in several ways (**Figure 1**). circRNAs can boost the expansion of polymerase II in the nucleus, enhancing their parent gene’s expression level. It can also recruit splicing bodies to promote reverse splicing, thus suppressing parent gene expression (6). The interaction between circRNAs and miRNAs is currently being studied intensively. By binding to specific sequences on miRNA targets, circRNAs inhibit miRNAs’ functions, regulating the miRNA-targeted genes expression. The relationship between circRNAs and microRNAs is currently the subject of most research. circRNAs restrict the actions of miRNAs by binding to certain target sequences, hence regulating the miRNA-targeted genes expression. It has also been demonstrated that some circRNAs contain open reading frames, which are translated into short peptides and control the translation of the specific mRNAs. CircRNAs influence cell growth, proliferation, and differentiation (7, 8) and can initiate, promote, and expedite the spread of cancer (9, 10). Consequently, they may serve as diagnostic and prognostic biomarkers and therapeutic targets (11, 12). This article will review the recent research progress and clinical value of circRNAs in NB.

## Carcinogenic circRNAs

### CircCUX1(circ_0132813)

CircCUX1 (circ_0132813) is the most studied circRNA in NB at present. It comes from the CUX gene located on chromosome 7 (chr7:101870650-101870949). Li originally investigated the mechanism of action and therapeutic potential of circCUX1 in NB (13). In this study, they discovered that circCUX1 was involved in aerobic glycolysis and the progression of NB. According to clinical data, circCUX1 was an independent risk factor for adverse outcomes in NB patients. Patients who exhibited high levels of circCUX1 had a decreased survival probability. CircCUX1 interacted with EWS RNA binding protein 1 (EWSR1) to stimulate MYC-related zinc finger protein (MAZ) trans-activation, resulting in CUX1 transcription modification and other tumor progression-related genes being transcribed. By inhibiting the interaction between Circux1 and EWSR1, the NB cell growth and invasion can be inhibited. As an alternate technique, the researchers designed an EWSR1 inhibitory peptide (EIP-22) comprising 22 amino acids that prevent the interaction between circCUX1 and EWSR. Using EIP-22, the researchers discovered that EWSR1 could prevent circCUX1-EWSR1 connections from reducing the development and migration of neuroblastoma SH-SY5Y cells. In animal experiments, mouse survival was improved by EIP-22 by reducing lung metastasis of SH-SY5Y cells.

According to Zhang and colleagues, circCUX1 was associated with NB cell proliferation, migration, invasion, and glycolysis (14). miR-16-5p is the direct target of circCUX1. Studies have demonstrated that miR-16-5p targets MYCN mRNA and inhibits tumor progression in NB (15). miR-16-5p expression was inhibited by high circCUX1 expression. Further research revealed that, in NB cells, DMRT2 was the target of miR-16-5p; the DMRT2 effects on the tumor cells proliferation, migration, invasion, and glycolysis can be reversed by miR-16-5p overexpression. Therefore, circCUX1 can promote NB cell proliferation, migration, invasion, and glycolysis by miR-16-5p/DMRT2 axis. In a similar mechanism of action, Fang et al. found that circCUX1 works through the miR432-5p/NOL4L axis (16), and Wang found that circCUX1 works through the miR-338-3p/PHF20 axis (17).

Unlike the first two mechanisms, Yang discovered the mechanism of circCUX1 in NB metabolism reprogramming (18). They found a protein sub-type consisting of 113 amino acids encoded by the CUX1 gene (p113). Additional validation tests showed that nuclear p113 encoded by circCUX1 increased NB cell proliferation, invasion, metastasis, lipid metabolism reprogramming, and mitochondrial activity. The transcriptional regulatory complex formed by P113 Zuotin-related factor 1 (ZRF1), and bromodomain protein 4 (BRD4) upregulated aldehyde dehydrogenase 3 family member A1 (ALDH3A1), NADH: ubiquinone oxidoreductase subunit A1 (NDUFA1) and NADH: ubiquinone oxidoreductase complex assembly factor 5 (NDUFAF5), by transactivation of ZRF1/BRD4. Therefore, these genes enhanced lipid metabolism (the production and oxidation of fatty acids) and mitochondrial activity. By inhibiting the interaction of p113-ZRF1, inhibitory peptides can impede cancer cell growth and invasion. High expression of p113, ZRF1, or BRD4 was associated with a reduced survival rate among NB patients. Based on these findings, it was hypothesized that circCUX1 subtypes encoded by p113 subtypes increase tumor growth by transactivating ZRF1/BRD4.

### CircKIF2A (circ_0129276)

CircKIF2A was first discovered by Ebrahim Mahmoudi’s team (19), and then the expression level and function of circKIF2A in NB tissues and cell lines were investigated by Yang et al. It was discovered that circKIF2A contributed to the proliferation and invasion of malignant cells. CircKIF2A may facilitate a more efficient glycolysis rate and energy metabolism in tumor cells, hence promoting their growth and function. According to further research, circKIF2A was activated through the miR129-5p/PLK4 axis (20). Moreover, Jin’s group discovered that circKIF2A was associated with elevated NB tissue levels and that circKIF2A mediated its activity by miR-377-3p/PRPS1 (21).

### CircDGKB (circ_0133622)

Yang observed that circDGKB was upregulated in NB tissue relative to normal ganglia and that there was a negative correlation between circDGKB expression levels and survival rates of NB patients. CircDGKB overexpression caused NB cells to proliferate, migrate, invade, and tumorigenesis while reducing their apoptosis. According to the luciferase reporter gene study, this enhancement of NB cells was accomplished by reducing miR-873 expression and increasing GLI1 (glioma-associated oncogene 1) gene expression. GLI1 is an important transcription factor of the Hedgehog signaling pathway, which is involved in the tumor progression of various tumors (22), including NB. circDGKB appears to be a promising possibility for new therapies and diagnostic markers of NB, according to this study (23).

### CircAGO2(Circ_0135889)

Argonaute 2 (AGO2) is the core component of microRNA-induced silencing complex. It plays a crucial function in microRNA-mediated gene silencing and significantly affects tumor development and progression. CircAGO2 comes from the AGO2 coding gene. circAGO2 is upregulated in numerous tumor tissues and cell lines, including gastric cancer, prostate cancer, colorectal cancer, and NB (24). There was a correlation between cancer prognosis and circAGO2 expression levels (24). Researchers have found that circAGO2 physically interacts with the human antigen R protein (HuR), activating and enriching it in the 3’-untranslated region of the target gene. Therefore, AGO2 binding is reduced, and microRNA-mediated gene silencing associated with cancer progression is inhibited. Using shRNA targeting circAGO2 could block downstream target gene activity and prevent the occurrence and growth of transplanted tumors in nude mice. Additionally, blocking circAGO2 and HuR connections with cell penetration inhibitory peptides will stop cancer cells from spreading and becoming invasive. These results indicated that circAGO2 facilitated tumor progression by blocking the inhibitory function of the key molecule HuR of the AGO2/microRNA complex.

### CircACAP2

CircACAP2 was discovered for the first time in breast cancer research; it can increase tumor growth and metastasis (25). Subsequently, Zhu and colleagues found that circACAP2 and hexokinase 2 (HK2) abundance in NB tissues and cell lines increased significantly (26). By suppressing circACAP2, migration, invasion, and glycolysis of NB cells were reduced, and apoptosis was induced. According to bioinformatics analysis, a luciferase reporter assay, and an RNA pull-down experiment, circACAP2 was identified as a target of miR-143-3p. In addition, the glycolytic enzyme HK2 was a direct target of miR-143-3p in NB cells. Thus, circACAP2 promotes tumor formation through the miR-143-3p/HK2 signal cascade. Moreover, the glycolytic enzyme HK2 was a direct target of miR-143-3p in NB cells. Thus, circACAP2 promotes tumor formation by the miR-143-3p/HK2 signal cascade.

### CircDLGAP4

CircDLGAP4 was first implicated with nervous system diseases, including ischemic stroke (27) and Parkinson’s disease (28). With the deepening of research, researchers gradually discovered its role in tumors (29) and nerve injury (30). Tan et al. investigated the role of exosome-delivered circDLGAP4 in the chemoresistance of NB cells (29). It was observed that doxorubicin-resistant cells showed HK2 higher expression and enhanced glycolysis. CircDLGAP4 delivered by the exocrine body promoted glycolysis, proliferation, and invasion of sensitive NB cells by regulating miR-143 and HK2 and established a new relationship between drug resistance and circDLGAP4/miR-143/HK2 axis in drug-resistant NB.

### Circ_0013401

Zhu’s team investigated the role of circ_0013401 in NB (31). They found that circ_0013401 expression increased in NB tissues. Circ_0013401 can accelerate tumor growth and metastasis in NB and inhibit tumor apoptosis and autophagy. This is accomplished by competitively binding miR-195, thus reducing its silencing inhibition on the target gene p21-activated kinase 2. (PAK2). The PAK2 increased expression contributes to the malignant biological behavior of NB tumor cells.

### CircPDE5A (circ_0002474)

Chen et al. reported the molecular role of circPDE5A in the NB malignant progression (32). In their investigation, circPDE5A expression was higher in NB tissues and cells. When circPDE5A is silenced, NB cells *in vitro* are inhibited from proliferating, migrating, invading, and metabolizing glycogen, and tumors *in vivo* are reduced in size. Additionally, by binding to miR-362-5p, circPDE5A regulates miR-362-5p’s effects on the malignant growth of NB *in vitro*, resulting in nucleolar protein 4-like (NOL4L) overexpression.

NOL4L has been reported to be an oncogene in multiple cancers, including NB (33). By altering the miR-362-5p/NOL4L pathway, the knockdown of circPDE5A at least partially inhibits the malignant evolution of NB, suggesting circPDE5A as a potential therapeutic target for NB and offering some evidence for its usage.

### Circ0125803

Tang et al. reported the molecular role of circ0125803 in NB proliferation and metastasis (34). They used high-throughput chip analysis technology to analyze the circular RNA expression in NB tissue. It was found that circ0125803 was a highly upregulated circRNA in NB samples. circ0125803 inhibition significantly reduced the proliferation and invasion rate of NB. Circ0125803 promotes the NB progression by blocking miR-197-5p and increasing E2F1 expression.

### CircHIPK3

CircHIPK3 has been confirmed to increase expression in various malignant tumors and promote tumor progression (35). However, there are few studies in NB. Wei Jiabin’s team studied the expression of circHIPK3 in NB cells and its role in NB progression (36). The effects of circHIPK3 on the proliferation, migration, and invasion of NB cells were detected by qRT-PCR, CCK-8, plate cloning test, scratch test, and transwell invasion test. It was confirmed that circHIPK3 was highly expressed in NB cells, and silencing circHIPK3 could inhibit the proliferation, migration, and invasion of NB cells. This study did not investigate how circHIPK3 promotes tumor cell proliferation, migration, and invasion; however, it gave some evidence for circHIPK3’s participation in NB.

### Circ0075829

Ren Dong and his colleagues first studied the Circ0075829 expression in NB and its role in cell proliferation and migration (37). The circ0075829 expression was increased in NB tumor tissues and cell lines. After the circ0075829 deletion, the proliferation and migration of NB cell lines were boosted, and ERK phosphorylation activity was triggered, but p38 and JNK phosphorylation activities were unaffected. The circ0075829 expression was negatively correlated with the INSS stage and risk stage and positively correlated with the total survival period of patients. The study found that circ0075829 can inhibit NB cell proliferation and migration. Its high expression is well correlated with clinical phenotype. It may play a role by stimulating the ERK/MAPK pathway and is a potential NB biomarker and treatment target.

### CircNHSL1

Liu Jingdong and colleagues discussed the circNHSL1 expression in NB and the role of chemotherapy tolerance (38). They found that the circNHSL1 expression in NB was higher than in normal tissues. The circNHSL1 expression in chemotherapy-resistant children and drug-resistant cell lines was higher than in chemotherapy-sensitive children. In chemotherapy-resistant cells, after silencing circNHSL1, the cell proliferation activity decreased, while the apoptosis rate increased, TGF- β The secretion level and expression level and the downstream expression level of p-Smad2 and p-Smad3 decreased. Donor recombinant TGF- β After treatment of circNHSL1 silenced drug-resistant cells, the cell proliferation activity increased, while the apoptosis rate decreased, and the drug-resistant characteristics were restored. CircNHSL1 may trigger the TGF-β/Smad signaling pathway associated with chemotherapy tolerance. Nonetheless, this report lacks extensive research on the circNHSL1 mechanism.

## Cancer suppressor circRNA

### CircRNA-TBC1D4, circRNA-NALAD2c and circRNA-TGFBR3

The research on circRNAs in NB is mostly related to promoting tumor progression. Lin and team members have found several possible tumor suppressor circRNAs (39). Five pairs of NB and adjacent normal fetal adrenal medulla samples were collected for high-throughput sequencing. The putative circRNAs were discovered by real-time quantitative reverse transcriptase polymerase chain reaction after bioinformatic analysis of the differentially expressed circRNAs host gene. Furthermore, an analysis was conducted of the clinical characteristics of NB and the key circRNAs.

Additionally, these key circRNAs were overexpressed in NB cell lines to investigate their biological functions. Consequently, 4704 differentially expressed circRNAs were discovered, including 2462 upregulated and 2242 downregulated circRNAs. As miR-21 is vital in the progression of NB, they further experimented with the circRNAs associated with miR-21. Numerous circRNAs were identified based on target prediction, including circRNA-TBC1D4, circRNA-NAALAD2, and circRNA-TGFBR3. Further study revealed that circRNA-TBC1D4, circRNA-NAALAD2, and circRNA-TGFBR3 were associated with clinical features. The expression of the three circRNAs in NB tissue was significantly lower than in normal adrenal tissue. The circRNA-TBC1D4 expression was related to the number of MYCN and lactate dehydrogenase concentrations.

Similarly, circRNA-NAALAD2 expression correlated with LDH concentration, while circRNA-TGFBR3 expression correlated with histological classification. A positive effect of circRNA-TBC1D4 overexpression on NB cell migration was seen *in vitro*, but it did not promote NB cell proliferation or colony formation. In conclusion, this study offered some evidence for the protective function of circRNAs in NB, suggesting that circRNAs may become an additional therapy option for NB.

### CircRNA functions and mechanism unclear

Zhang and colleagues (40) downloaded the RNA-seq data of 39 NB and 2 normal cell lines from the Sequence Read Archive (SRA) database. They described the comprehensive characterization of circRNAs in NB Cell Lines. They discovered 29 circRNAs with altered expression levels. After constructing ceRNA networks, hsa_circ_0005379, hsa_circ_0002343, and hsa_circ_0001361 were identified as critical regulators in the ceRNA networks, which might regulate PI3K/Akt/mTOR signaling pathway and epithelial-mesenchymal transition (EMT). However, no more mechanistic investigations have been conducted on how these circRNAs affect signaling pathways and EMT processes.

## Discussion

Non-coding RNAs (ncRNAs) are crucial in the pathogenesis of neuroblastoma (NB). In recent years, circRNAs have emerged as an important ncRNA in NB research, with increasing studies demonstrating their involvement in tumor cell proliferation, invasion, and migration. One of the key discoveries of prior research is that circRNAs are differentially expressed in NB relative to normal tissue, suggesting that they may play a role in the pathogenesis of the disease. **Table 1** summarizes the current research and the role of circRNAs in NB. Like lncRNAs and miRNAs, circRNAs exhibit a dual function in promoting tumor progression and inhibiting tumor development. Down-regulating the expression of upregulated circRNAs inhibits tumor growth, highlighting their potential as therapeutic targets.

**Table 1:** Role of circRNAs in NB.

CircRNAs	Expression	Functions in NB	Potential mechanism	Reference
CircCUX1 (Circ_0132813)	Upregulated	1. Promotes aerobic glycolysis and NB proliferation and invasion	1. CircCUX1 combines with EWSR1 to promote its interaction with MAZ protein, resulting in the trans-activation of MAZ and the transcription change of CUX1 and other tumor progression-related genes	(13)
		2. Promotes the proliferation and invasion of tumor cells, and induce glycolysis metabolism	2. Through miR-16-5p/DMRT2 axis, miR432-5p/NOL4L axis and miR-338-3p/PHF20 axis	(14, 16, 17)
		3. Promotes lipid metabolism reprogramming, mitochondrial activity, proliferation, invasion and metastasis of NB cells	3. p113 encoded by circCUX1 promotes tumor progression *via* trans-activation of ZRF1/BRD4	(18)
CircKIF2A (Circ_0129276)	Upregulated	Promotes the proliferation and invasion of tumor cells and improve the glycolysis level of tumor cells	Through miR129-5p/PLK4, miR-377-3p/PRPS1 axis	(20, 21)
CircDGKB (Circ_0133622)	Upregulated	Promotes the proliferation, migration, invasion and tumorigenesis of NB cells, and reduce cell apoptosis	Via miR-873/GLI1 axis	(23)
CircAGO2 (Circ_0135889)	Upregulated	Promotes the growth and invasion of NB tumor cells	CircAGO2 interacts with HuR protein to regulate the function of AGO2/miRNA complex	(24)
CircACAP2	Upregulated	Promotes the migration and invasion of cancer cells and inhibit the apoptosis of cancer cells	CircACAP2 acts through the miR-143/HK2 axis	(26)
CircDLGAP4	Upregulated	Promotes the proliferation and invasion of cancer cells, induce glycolysis, and induce drug resistance	CircDLGAP4 works through the miR-143/HK2 axis	(29)
Circ_0013401	Upregulated	Induces tumor growth and metastasis, prevent tumor cell apoptosis and autophagy	Circ_0013401 works through the miR-195/PAK2 axis	(31)
CircPDE5A (Circ_0002474)	Upregulated	Promotes the proliferation and invasion of tumor cells, and induce glycolysis metabolism	CircPDE5A works through miR-362-5p/NOL4L axis	(32)
Circ0125803	Upregulated	Promotes tumor proliferation and invasion	Circ0125803 works through the miR-197-5p/E2F1 axis	(34)
CircHIPK3	Upregulated	Promotes tumor proliferation and invasion, promote NB cell proliferation, migration and invasion	Further mechanism study not performed.	(36)
Circ0075829	Upregulated	Enhances the proliferation and migration of NB cells	It may work by activating ERK/MAPK pathway	(37)
CircNHSL1	Upregulated	Promotes the proliferation of NB cells, inhibit the rate of apoptosis, and induce drug resistance	It is possible to activate TGF- β/Smad signal pathway participates in chemotherapy tolerance	(38)
CircRNA-TBC1D4,	Downregulated	Inhibits the proliferation and migration of cancer cells	It may weaken the silencing of target genes by miRNA-21 by competing with miRNA-21	(39)
CircRNA-NAALAD2	Downregulated	Inhibits the proliferation and migration of cancer cells	It may weaken the silencing of target genes by miRNA-21 by competing with miRNA-21	(39)
CircRNATGFBR3	Downregulated	Inhibits the proliferation and migration of cancer cells	It may weaken the silencing of target genes by miRNA-21 by competing with miRNA-21	(39)
Circ_0005379	Unknown	Unclear	As a critical regulator in the ceRNA networks formed by 13 genes	(40)
Circ_0002343	Unknown	Unclear	It may work by regulating PI3K/Akt/mTOR signaling *via* RAC1	(40)
Circ_0001361	Unknown	Unclear	Possibly involved in EMT	(40)

EWSR1, EWS RNA binding protein 1; MAZ, MYC-related zinc finger protein; DMRT2, Doublesex and mab-3 related transcription factor 2; NOL4L, Nucleolar protein 4 like; PHF20, PHD Finger Protein 20; ZRF1, Zuotin-related factor 1; BRD4, bromodomain protein 4; PLK4, Polo-like kinase 4; PRPS1, Phosphoribosyl pyrophosphate synthetase 1; GLI1, Glioma-associated oncogene 1; AGO2, Argonaute 2; HK2, hexokinase 2; PAK2, P21-activated kinase 2; HuR, Human antigen R; RAC1, Rac family small GTPase 1; EMT, epithelial mesenchymal transition.

Conversely, some down-regulated circRNAs exhibit protective effects in NB and may serve as potential drug targets for treating the disease. Other studies have demonstrated the potential of circRNAs as diagnostic biomarkers for NB patients, particularly in blood samples. However, most circRNA research has centered on their sponge adsorption and competitive binding to miRNAs to counteract the miRNA-mediated silence of target genes. Relatively less is known about additional action methods, such as the peptide encoded by circRNAs. A more comprehensive investigation of circRNAs would enhance our understanding of their function in NB and provide broader research avenues for NB diagnosis and treatment.

In addition to their potential as biomarkers, circRNAs have been demonstrated to modulate several signaling pathways involved in NB development and progression. For example, a study by Liu et al. (38) found that the circNHSL1 promoted NB cell proliferation, inhibited the apoptosis rate, and induced drug resistance by activating the TGF- β/Smad pathway. Similarly, a study by Tang et al. (34) identified a circRNA, Circ0125803, that promoted NB cell proliferation and invasion by targeting the miR-197-5p/E2F1 axis.

Despite these findings, there are still gaps and contradictions in the research that require attention. For instance, the functional roles of several circRNAs in NB are poorly understood, as are their mechanisms of action. Furthermore, the circRNAs expression patterns can vary widely between different studies, which may be due to differences in sample size, tissue source, and analysis methods. More comprehensive studies that use larger sample sizes and standardized methods for circRNA detection and analysis are needed to better understand the role of circRNAs in NB.

In summary, previous studies have identified several circRNAs differentially expressed in NB and have been shown to regulate various signaling pathways involved in the disease’s development and progression. Additional research is required to completely comprehend the physiological roles of circRNAs in NB and their potential as diagnostic and therapeutic targets.

## Author contributions

CY and JT conceived the scope of the manuscript. CY and KW wrote the manuscript. All authors discussed the manuscript and made comments on the manuscript. All authors contributed to the article and approved the submitted version.
